# iDINGO—integrative differential network analysis in genomics with *Shiny* application

**DOI:** 10.1093/bioinformatics/btx750

**Published:** 2017-11-29

**Authors:** Caleb A Class, Min Jin Ha, Veerabhadran Baladandayuthapani, Kim-Anh Do

**Affiliations:** Department of Biostatistics, The University of Texas MD Anderson Cancer Center, Houston, TX, USA

## Abstract

**Motivation:**

Differential network analysis is an important way to understand network rewiring involved in disease progression and development. Building differential networks from multiple ‘omics data provides insight into the holistic differences of the interactive system under different patient-specific groups. DINGO was developed to infer group-specific dependencies and build differential networks. However, DINGO and other existing tools are limited to analyze data arising from a single platform, and modeling each of the multiple ‘omics data independently does not account for the hierarchical structure of the data.

**Results:**

We developed the iDINGO R package to estimate group-specific dependencies and make inferences on the integrative differential networks, considering the biological hierarchy among the platforms. A *Shiny* application has also been developed to facilitate easier analysis and visualization of results, including integrative differential networks and hub gene identification across platforms.

**Availability and implementation:**

R package is available on CRAN (https://cran.r-project.org/web/packages/iDINGO) and *Shiny* application at https://github.com/MinJinHa/iDINGO.

**Supplementary information:**

Supplementary data are available at *Bioinformatics* online.

## 1 Introduction

The analysis of differential networks has led to a deeper understanding of network rewiring, which explains changing molecular relationships associated with a characteristic of interest, such as disease states or progression, clinical treatments or environmental stress (Bandyopadhyay *et al.*, 2010; Califano, 2011). Most of the previous approaches for differential network analysis have relied on different correlation-based metrics to measure the dependencies between pairs of nodes in a network (Hudson *et al.*, 2009; Reverter *et al.*, 2006). However, these methods are limited to marginal correlation networks (i.e. two nodes at a time) that are estimated separately using observations within each group, and generally do not consider relationships that are conserved across multiple groups. This has been refined in the DINGO framework (Ha *et al.*, 2015) that separates group-specific conditional dependencies into global and group-specific components, and this method has been shown to improve performance over other existing methods in simulation studies and with real data.

At the same time, integromic analyses (including genomics, epigenomics, proteomics and others) have provided biological and clinical insight into a variety of diseases (Gerstung *et al.*, 2015; Qin, 2008). Differential network analysis of integromic datasets introduces new opportunities, as an understanding of the relationships of elements across platforms can provide a more complete biological understanding of the characteristic of interest. Our integrative approach identifies a set of edges between nodes that are differentially connected between patient groups, including directed edges between platforms and undirected edges within platforms. Using data from additional platforms allows us to adjust for the upstream data, providing a more refined network than the original DINGO method. The resulting network allows us to identify ‘hub nodes’ (nodes with the greatest number of outgoing or undirected edges) across platforms, which may have the greatest effect on the clinical/grouping variable (Flintoft, 2004).

In this paper, we present the R package ‘iDINGO’ (with accompanying *Shiny* application) as an expansion of the ‘DINGO’ package. This package integrates relationships between different ‘omics levels in the analysis using a chain graph model. Parallelization is implemented to improve computation time, and a multiple-testing correction is also included to improve inference on differential edges. Finally, we introduce a *Shiny* application to facilitate easier analysis and visualization.

## 2 The iDINGO R package

We can integrate ordered data platforms using the chain graph model. For example, we can integrate microRNA, mRNA and protein data using the assumed ordering,

microRNA<mRNA<Protein,

which means that microRNA can affect mRNA and protein, and mRNA can affect protein, but not vice versa. In this case, we have a set of nodes $V=V_{M}\cup V_{R}\cup V_{P}$, where $V_{M}=\left\{M_{1},M_{2},\ldots \}\right\}$ is a set of microRNA nodes, $V_{R}=\{R_{1},R_{2},\ldots \}$ is a set of mRNA nodes and $V_{P}=\{P_{1},P_{2},\ldots \}$ is a set of protein nodes, and a set of edges E that may contain both directed (→) and undirected (-) edges between and within $V_{M}$, $V_{R}$ and $V_{P}$, respectively. Following the Markov property for chain graphs (Frydenberg, 1990; Lauritzen and Wermuth, 1989), the within- and between-platform conditional independence is defined as follows:

$${\left(D1\right)\;M}_{i}⫫M_{j}|\;V_{M}\setminus \{M_{i},M_{j}\}$$

$$\left(D2\right)\;M_{i}⫫R_{j}|\;V_{M}\cup V_{R}\setminus \{M_{i},R_{j}\}$$

$${\left(D3\right)\;R}_{i}⫫R_{j}|\;V_{M}\cup V_{R}\setminus \{R_{i},R_{j}\}$$

$$\left(D4)\;M_{i}⫫P_{j}|\;V\setminus \{M_{i},P_{j}\right\}$$

$$\left(D5)\;R_{i}⫫P_{j}|\;V\setminus \{R_{i},P_{j}\right\}$$

$$(D6)\;P_{i}⫫P_{j}|\;V\setminus \{P_{i},P_{j}\},$$

where the conditional dependencies in (D1), (D3) and (D6) encode undirected edges within microRNA, mRNA and protein, respectively, and those in (D2), (D4) and (D5) represent directed edges microRNA→mRNA, microRNA→protein and mRNA→protein. In our iDINGO framework, we investigate the differential network between those integrative dependencies for random variables from multiple platforms, which follows a multivariate normal distribution. This approach allows us to consider more refined biological relationships between platforms than the original DINGO package, which did not consider dependencies between different data platforms (the implementation of this chain graph model is described in Supplementary Section S1, along with a notation table).

The input to iDINGO includes up to three matrices containing expression data on the same samples from different platforms, as well as a vector denoting the group membership of each sample. The final iDINGO object contains all of the possible edges among and between platforms, along with their respective partial correlations for each group, differential scores and *P*-values. The iDINGO methodology is general to be applied with just one, two or more than three platforms with known ordering information between them (in the case of one platform, the regular DINGO algorithm will be used).

Examples of the iDINGO implementation have been provided in Supplementary Section S5. In addition, we discuss additional features added to iDINGO, including parallelization, false discovery rate (FDR) corrected *P*-values and differential network plotting (Supplementary Section S1).

## 3 *Shiny* web application

iDINGO has been implemented in a web application using the *Shiny* R package (Chang *et al.*, 2017), to provide a user-friendly integromic analysis method. A description of its graphical user interface and a usage example are provided in Supplementary Section S4.

One, two or three omics datasets (matched samples) are to be provided as text files. The sample group classifiers are input as another text file, containing the binary group information for the samples. We recommend generating pathway-based iDINGO networks with no more than a few hundred elements, due to the computational resources and time required. More information about pathway-level analysis is provided in Supplementary Section S2, as well as other considerations in platform integration in Supplementary Section S3.

**Fig. 1:**
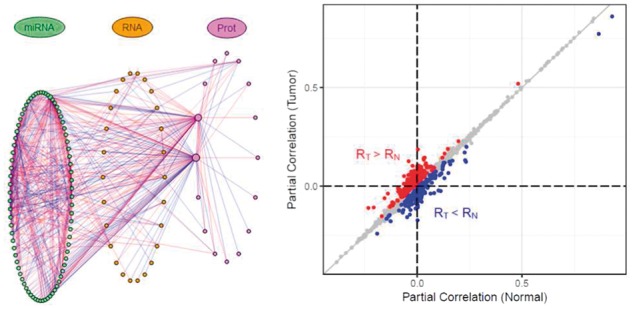
*Left*: iDINGO differential network plot (with microRNA nodes on the left, mRNA nodes in the center and protein nodes on the right), containing partial correlations increased in breast cancer (red edges) or normal tissue (blue edges), with FDR < 0.1. *Right*: A scatterplot comparing the partial correlations of the edges in the two groups, colored as in the network

After running iDINGO, the resulting network is presented in the main panel along with a table of the top hub elements (those with the greatest number of differential edges). In the network plot (Fig. 1), nodes are colored by platform level, and the differential network can be depicted in multiple layouts. The differential score threshold can be set to further filter which edges are considered ‘differential’ and included in the plotted network. A scatterplot of group-specific partial correlations (Fig. 1) is provided to compare the magnitude of the group-specific dependencies.

## Funding

This work was supported by the National Institutes of Health [R01-CA194391 and P50-CA070907-18 to M.J.H. and V.B., R01160736 to V.B. and P30-CA016672 to V.B. and K.A.D.], the National Science Foundation [1463233 to V.B.] and the Moon Shot Grant Program at MD Anderson Cancer Center [to C.A.C. and K.A.D.].

*Conflict of Interest*: none declared.

## Supplementary Material

Supplementary Data
