# Supplementary material for: iDINGO—integrative differential network analysis in genomics with Shiny application
Source: Bioinformatics. 2017 Nov 29;34(7):1243–5. doi: 10.1093/bioinformatics/btx750 (PMC6030922; doi:10.1093/bioinformatics/btx750)
Supplement: Supplementary Data [file btx750_suppinfo-v7-final.docx]

Supplementary Material

**iDINGO: Integrated Differential Network Analysis with Shiny Application**

Caleb A. Class^1^, Min Jin Ha^1,*^, Veerabhadran Baladandayuthapani^1^, and Kim-Anh Do^1^

^1^Department of Biostatistics, The University of Texas MD Anderson Cancer Center, Houston, TX 77030, USA

Contents

[Section S1: iDINGO Implementation in *R* 1](#_Toc497128732)

[Section S1.1 Additional features in iDINGO R package 2](#_Toc497128733)

[Section S1.2 Table of Notations 2](#_Toc497128734)

[Section S2: Pathway analysis in iDINGO 2](#_Toc497128735)

[Section S3: Advanced platform integration 3](#_Toc497128736)

[Section S4: *Shiny*-iDINGO application interface 4](#_Toc497128737)

[Section S5: Examples using TCGA Breast Cancer Data 6](#_Toc497128738)

[Section S6: Speed-Up with parallel bootstrapping 7](#_Toc497128739)

[Section S7: Effect of Sample Size 8](#_Toc497128740)

[Section S8: Supplemental References 9](#_Toc497128741)

# Section S1: iDINGO Implementation in *R*

The Markov properties applied to the chain graph model allows the three independent DINGO analyses (with the assumed ordering of *microRNA < mRNA < Protein* for this example) as follows:

(S1) DINGO will run with microRNA data only, to identify differential microRNA-microRNA relationships from the conditional independence (D1).

(S2) DINGO will then run using the combined microRNA-mRNA data set, retaining only edges on microRNA->mRNA and mRNA-mRNA edges from the conditional independencies in (D2) and (D3), respectively.

(S3) Finally, the full combined data set will be analyzed using DINGO to identify differential edges on microRNA->protein, mRNA-> protein and protein-protein from the conditional independencies in (D4), (D5) and (D6), respectively.

Groupwise partial correlations, differential scores, p-values for all of the possible edges are returned as the final iDINGO result. This can also be run for one, two, or extended to more than three different platforms with known connectivity.

iDINGO is a data-driven de novo method, and it will consider all possible edges between all of the nodes provided in the input matrix/matrices. Because of this, it is preferred to conduct this differential network analysis at a pathway level to identify more potentially relevant biological differences between groups. We discuss this in the Section S2: “Pathway analysis in iDINGO.” In addition, more complicated cases that do not entirely fit the platform1 < platform2 < platform3 ordering are discussed in “Advanced platform integration.”

## Section S1.1 Additional features in iDINGO R package

- Parallelization – The bootstrapping step is the most computer- and time-intensive step of the DINGO and iDINGO process, using over 95% of the computation time for a run with the recommended minimum 100 bootstraps. Parallel bootstrapping has been implemented in our iDINGO package, using the R parallel package (R Core Team, 2016). This speeds up performance dramatically, as shown in the section “Speed-Up with parallel bootstrapping.”
- False discovery corrected p-values – We adapt an Empirical Bayes approach to build differential networks by thresholding differential scores as implemented in the R package GGMridge (Efron, 2004; Ha and Sun, Wei, 2014). These guide us to use more informative cutoffs in defining differential networks for both DINGO and iDINGO.
- Network plotting – The *plotNetwork* function allows you to plot the result of a DINGO or iDINGO run, specifying a differential score or p-value to use as a threshold for edge inclusion. This function uses the *visNetwork* and *igraph* libraries, and *igraph* layouts can be specified for the plot (Almende B.V. et al., 2017; Csardi and Nepusz, 2006).

## Section S1.2 Table of Notations

| Symbol | Meaning |
| --- | --- |
| $V$ | a set of all nodes from all three platforms at microRNA, mRNA and protein levels |
| $V_{M}$ | a set of microRNA nodes |
| $V_{R}$ | a set of mRNA nodes |
| $V_{P}$ | a set of protein nodes |
| $E$ | a set of edges for nodes in V, that includes undirected (within platform) and directed (between platforms) |
| $M_{i}$ | $i^{th}$ microRNA node in $V_{M}$ |
| $R_{i}$ | $i^{th}$ mRNA node in $V_{R}$ |
| $P_{i}$ | $i^{th}$ protein node in $V_{P}$ |

# Section S2: Pathway analysis in iDINGO

Due to the computational time required (see the section “Effect of Sample Size”), we recommend generating DINGO networks with no more than a few hundred elements. The recommended method to select a relevant subset of genes is to focus on a specific pathway or set of pathways—for example, a gene set or sets from the Molecular Signatures Database (Liberzon et al., 2011). This is relatively straightforward for most data types: an RNA-based expression matrix can be filtered to only include genes that are represented in a pathway of interest, and protein or methylation identifiers can be matched to gene symbols to accomplish the same purpose. This is simply done using *R*, and we have also provided the capability to do gene set filtering using *Shiny­*-iDINGO with a user-provided gene set database (see the section “*Shiny*-iDINGO application interface”). However, microRNA can have multiple RNA targets, so a slightly different approach is needed. We recommend the use of a microRNA-target gene database, such as that of Doecke *et al.* (Doecke et al., 2014). MicroRNA with known or computationally predicted relationships with genes in the pathway of interest can be included in the final iDINGO input set. Occasionally, this results in a large set of microRNA potentially affecting the pathway, so additional filtering steps, such as filtering by variance, may be useful. We provide example analyses in the section “Examples using TCGA Breast Cancer data.”

# Section S3: Advanced platform integration

Thoughtful consideration should be given when integrating different data platforms in iDINGO. In our example, the assumed order of *microRNA < RNA < Protein* will generally be correct, but a researcher may also want to consider other possible interactions (such as transcription factors, which are proteins that can affect mRNA expression levels). If one wanted to consider the effects of these proteins on mRNA in iDINGO, those proteins should be moved to the “mRNA” platform using the `cbind` command in *R*:

# The variable `tf` is either a list of transcription factors, or the column numbers that correspond to

# those transcription factors in the protein data matrix. If `tf` is a character vector, it must match

# exactly with the target column names.

tf <- c(“p53”, “Sp1”)

# Move transcription factors from protein matrix to the mRNA matrix.

dat.mRNA <- cbind(dat.mRNA, dat.protein[, tf])

# Remove transcription factors from the protein matrix.

dat.protein <- dat.protein[, !(colnames(dat.prot.dingo) %in% tf)]

This procedure can be used generally when a “downstream” element is expected to affect “upstream” elements: the downstream element should be moved forward to the upstream level. Similarly, if two full platforms are expected to affect each other and are not expected to have a particular ordering between them, they could be entirely combined using `cbind`.

# Section S4: *Shiny*-iDINGO application interface

The *Shiny-iDINGO* application currently includes three tabs: Job Setup, Visualize Results, and Download Results. These allow the user to run DINGO, view output figures and tables, and download result files.

The Job Setup tab is presented in Figure S1. Data input boxes are provided to input up to three ‘omics data sets, as text files containing p×n matrices, with sample names as the column names (this is different from in the DINGO *R* package itself, but we use it in the *Shiny* app as this is the format generally used in this kind of study). Optional names can be put for each of the data sets, such as “microRNA,” “RNA” and “Protein” as in the example below. The sample group classifiers are provided as a text file defining the binary group membership of each sample. Samples must be in the same order in all data sets and in the classification file. The number of bootstraps to complete for each model, as well as cores to use for parallel computing, should also be specified. 100 bootstraps is the recommended minimum, and parallel computation speeds up this process considerably.

Optional gene set filtering can also be conducted in the *Shiny* application using a user-provided .gmt or .rds file containing a list of gene sets, such as the .gmt files provided by MSigDB (Liberzon et al., 2011). The identifiers in the uploaded file will be used to filter genes from the omics data sets by row name, so the format of the gene names in the gene set file must match the format of the row names in the omics sets (i.e. gene symbol, Entrez ID, etc.). If a gene set file is uploaded, an additional drop-down box will appear containing the available gene sets for selection. Note that the “brca” example data provided with the *Shiny* application has already been filtered, so it does not require gene set filtering.

Next, an example of the Visualize Results tab is presented in Figure S2. The differential network plot allows the user to explore the differential network generated by DINGO/iDINGO. Only differential edges passing the inclusion threshold are provided in the plot (threshold can be by p-value or differential score, and this is set in the left panel). In our default layout option, nodes from different platforms are grouped together (leading to an overall cylindrical shape for a multi-platform network), but other layouts are possible. Nodes can be selected either by clicking on them or using the drop-down menu. Edges with a higher partial correlation in Group 2 are in red, otherwise they are blue. A scatterplot of the partial correlation for Group 2 vs. that of Group 1 is below the network plot, with each edge as one point. Significant edges are red and blue as above (set by the threshold on the left), and insignificantly different edges are grey. A table containing the “hub nodes” most connected to other nodes by differential edges is provided on the right.


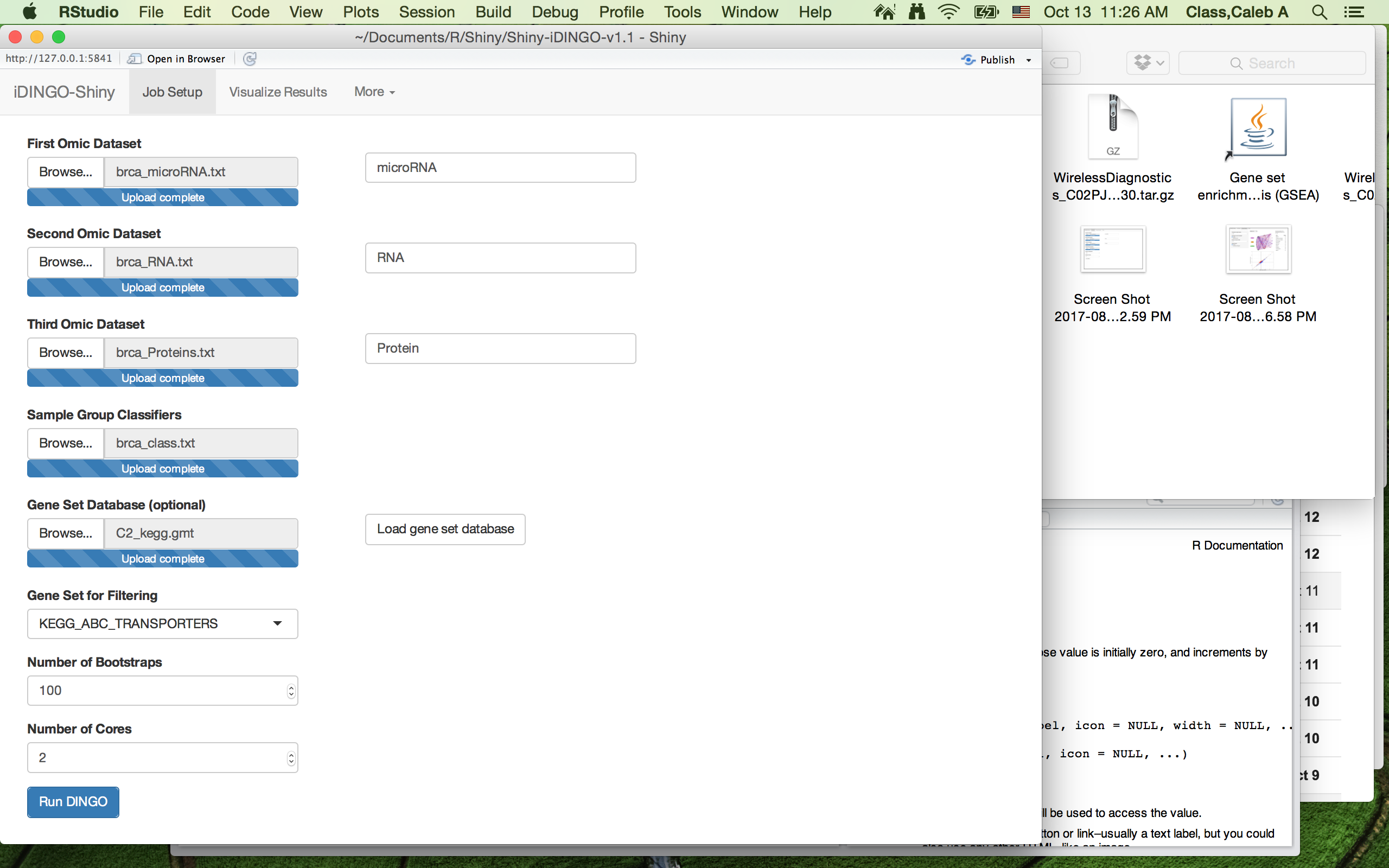


Figure S1. Shiny-iDINGO application: input interface. Includes platform data input, sample group classifier input, gene set filtering (optional), and iDINGO run options.


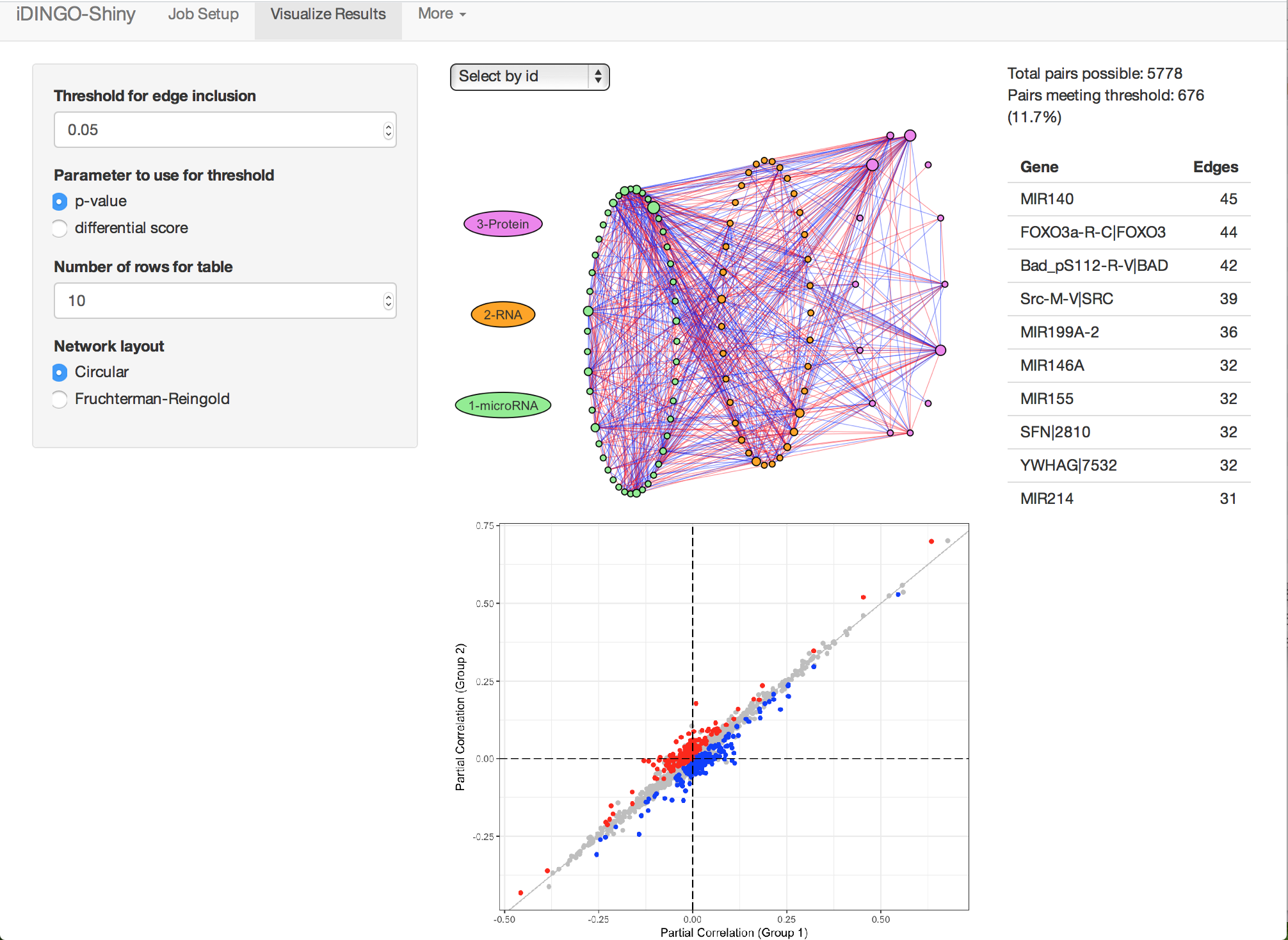


Figure S2. Shiny-iDINGO visualization interface. Includes differntial network plot, partial correlation scatterplot, and hub network table.

Finally, the Download Results tab provides a table or .rds file with results from DINGO/iDINGO.

# Section S5: Examples using TCGA Breast Cancer Data

A cohort of 627 breast cancer tissue samples and 26 adjacent normal samples (with available RNA-Seq, microRNA-Seq and RPPA data) were downloaded using TCGA-Assembler in R (Zhu et al., 2014). For this example, we chose to analyze the PI3K/Akt pathway from the Pathway Interaction Database, as well as the p53 pathway from Biocarta (Nishimura, 2001; Schaefer et al., 2009). Both gene sets were accessed through the MSigDB (Subramanian et al., 2005).

For the PI3K/Akt pathway 35 mRNA and 31 proteins were identified as members of this pathway. We used the validated microRNA-target gene database compiled by Doecke *et al.* to identify 128 microRNA that target at least one of the mRNA members of this pathway (Doecke et al., 2014). An additional filtering step required that microRNA, mRNA, and proteins be detected in at least 90% of the samples to be included in this analysis. This resulted in a dataset containing 102 microRNA, 35 mRNA, and 23 proteins. These were analyzed using iDINGO, with 100 bootstraps in parallel on 8 processors.

A plot of the microRNA-mRNA-Protein differential network is presented in Figure S3. microRNA nodes are green, mRNA nodes are orange, and protein nodes are pink. Red edges signify higher partial correlations between elements in breast cancer tumors, while blue edges signify higher partial correlations in normal tissue. GSK3 protein, which is important in breast cancer susceptibility (Aristizabal-Pachon and Castillo, 2017), was identified hub with the highest degree in the network, and its sub-network is highlighted in the second plot. As seen in the second plot, this protein is connected to most of the microRNA, mRNA, and proteins in the differential network.


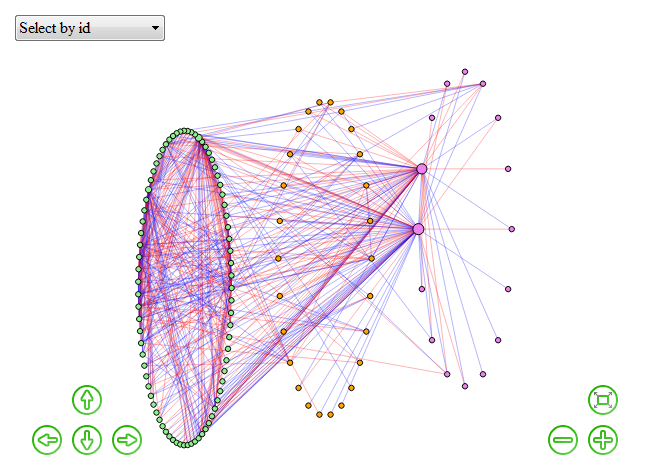

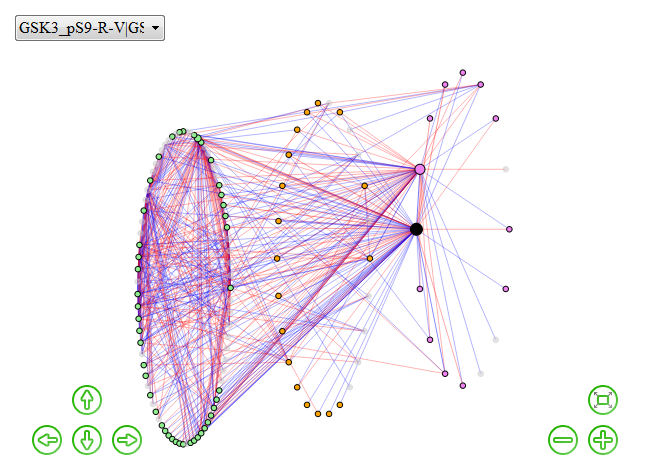
Figure S3. *Left:* Differential network between breast cancer tumors and adjacent normal tissue for PI3K/Akt pathway. *Right:* Same differential network, with hub protein GSK3 highlighted with its nearest neighbors.

For the p53 pathway, we identified 16 mRNA and 14 proteins as members, as well as 154 potential microRNA members using the microRNA-target database. We filtered for only elements that were detected in at least half of samples (plus an additional minimum variance of 1.5 for microRNA). The integromic data set analyzed using iDINGO contained 31 microRNA, 16 mRNA, and 10 proteins. One of these proteins, p53, is a known transcription factor, so it was moved to the mRNA level to allow it to affect both mRNA’s and proteins.

After running iDINGO, the differential network (p < 0.01) was plotted using the plotNetowork function, and the results are presented in Figure S4. 157 of 1596 possible edges (9.8%) were significantly differential with p < 0.01. Proteins PCNA and p21 were identified as top hubs, with 26 and 22 differential edges, respectively. p53 protein was also one of the top hubs (18 differential edges). The p53 sub-network is presented on the right in Figure S4, and it shows differential edges connecting with 7 microRNA, 2 mRNA, and 9 proteins. These include connections between p53 and ATM (RNA and protein), BAX (RNA and protein), and BCL2 (protein) which are in close proximity with p53 in the Biocarta network (Nishimura, 2001). The main hub, PCNA protein, has also been highly implicated in breast cancer (Beenken and Bland, 2002; Malkas et al., 2006). This protein is linked to CDK2, CDK4, and GADD45 RNA, all of which have known relationships with PCNA (Nishimura, 2001).


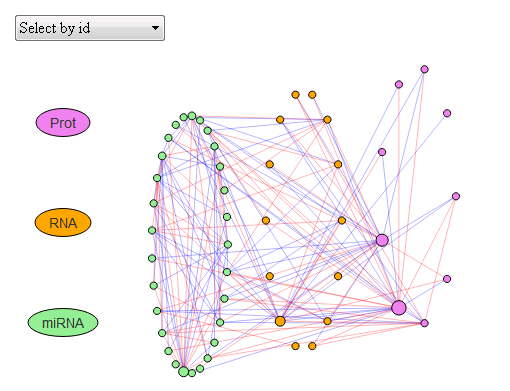

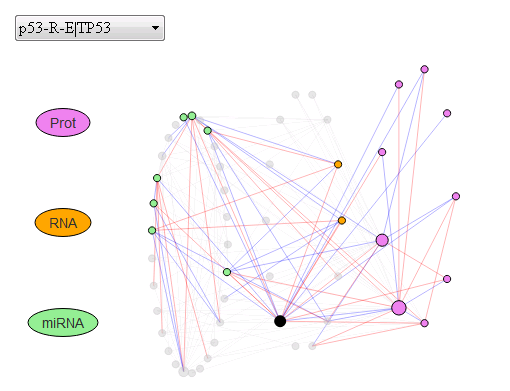


Figure S4. *Left:* Differential network between breast cancer tumors and adjacent normal tissue for p53 pathway. *Right:* Same differential network, with hub protein p53 highlighted with its nearest neighbors.

# Section S6: Speed-Up with parallel bootstrapping

Parallel bootstrapping was implemented in the iDINGO package, using the R parallel package (R Core Team, 2016). Performance was tested using a simulation of a 40×40 data set in standard DINGO, where the expression of the 40 genes is normally distributed and the 40 samples are evenly split between two groups. Each simulation was repeated 5 times, and the runtime was averaged to obtain each data point. As expected (and presented in Figure S5), overall runtime decreases exponentially with an increasing number of processors.

Figure S5. Simple simulation showing speed-up with parallel bootstrapping.

# Section S7: Effect of Sample Size

We also investigated the effect of the number of genes and samples on runtime in a normal DINGO run, using simulated data. For these runs, the samples are evenly split between the two groups to be differentiated. 100 bootstraps with 4 processors running in parallel were used in all simulations. Each #gene/#sample set was repeated 5 times, and the runtimes were averaged to obtain the results in the table below. We see runtimes near 2 hours with 300 genes and 100 samples in this parallel environment. This should be considered when running DINGO or iDINGO jobs, and networks containing larger amounts of elements can be built if computational resources and time permit. However, it is generally recommended to use smaller sets in the interactive *Shiny*-iDINGO application.

Table S1. Runtimes (seconds) for simulated DINGO runs with specified number of genes and samples (samples evenly split into 2 groups for DINGO analysis)

|  |  |  |  |  |
| --- | --- | --- | --- | --- |
|  | # Samples | | | |
| # Genes | 10 | 30 | 50 | 100 |
| 30 | 144.7 | 107.7 | 136.5 | 268.3 |
| 50 | 195.1 | 123.8 | 130.0 | 280.4 |
| 100 | 394.5 | 241.1 | 269.8 | 560.3 |
| 200 | 1709.2 | 1260.5 | 1398.2 | 2708.8 |
| 300 | 4933.9 | 4493.0 | 4700.6 | 7084.2 |

# Section S8: Supplemental References

Almende B.V., Thieurmel, B., and Robert, T. (2017). visNetwork: Network Visualization using “vis.js” Library.

Aristizabal-Pachon, A.F., and Castillo, W.O. (2017). Role of GSK3β in breast cancer susceptibility. Cancer Biomark. *18*, 169–175.

Beenken, S.W., and Bland, K.I. (2002). Biomarkers for breast cancer. Minerva Chir. *57*, 437–448.

Csardi, G., and Nepusz, T. (2006). The igraph software package for complex network research. InterJournal *Complex Systems*, 1695.

Doecke, J.D., Chekouo, T.C., Stingo, F., and Do, K.-A. (2014). miRNA Target Gene Identification: Sourcing miRNA Target Gene Relationships for the Analyses of TCGA Illumina MiSeq and RNA-Seq Hiseq Platform Data. Int. J. Hum. Genet. *14*, 17–22.

Efron, B. (2004). Large-Scale Simultaneous Hypothesis Testing. J. Am. Stat. Assoc. *99*, 96–104.

Ha, M.J., and Sun, Wei (2014). Partial Correlation Matrix Estimation Using Ridge Penalty Followed by Thresholding and Re-estimation. Biometrics *70*, 765–773.

Liberzon, A., Subramanian, A., Pinchback, R., Thorvaldsdóttir, H., Tamayo, P., and Mesirov, J.P. (2011). Molecular signatures database (MSigDB) 3.0. Bioinformatics *27*, 1739–1740.

Malkas, L.H., Herbert, B.S., Abdel-Aziz, W., Dobrolecki, L.E., Liu, Y., Agarwal, B., Hoelz, D., Badve, S., Schnaper, L., Arnold, R.J., et al. (2006). A cancer-associated PCNA expressed in breast cancer has implications as a potential biomarker. Proc. Natl. Acad. Sci. U. S. A. *103*, 19472–19477.

Nishimura, D. (2001). BioCarta. Biotech Softw. Internet Rep. *2*, 117–120.

R Core Team (2016). R: A Language and Environment for Statistical Computing (Vienna, Austria: R Foundation for Statistical Computing).

Schaefer, C.F., Anthony, K., Krupa, S., Buchoff, J., Day, M., Hannay, T., and Buetow, K.H. (2009). PID: the Pathway Interaction Database. Nucleic Acids Res. *37*, D674-679.

Subramanian, A., Tamayo, P., Mootha, V.K., Mukherjee, S., Ebert, B.L., Gillette, M.A., Paulovich, A., Pomeroy, S.L., Golub, T.R., Lander, E.S., et al. (2005). Gene set enrichment analysis: A knowledge-based approach for interpreting genome-wide expression profiles. Proc. Natl. Acad. Sci. *102*, 15545–15550.

Zhu, Y., Qiu, P., and Ji, Y. (2014). TCGA-Assembler: open-source software for retrieving and processing TCGA data. Nat. Methods *11*, 599–600.

TP53 Gene - GeneCards | P53 Protein | P53 Antibody.
